# Synthesis, characterization, quantum chemical calculations and anticancer activity of a Schiff base *NNOO* chelate ligand and Pd(II) complex

**DOI:** 10.1371/journal.pone.0231147

**Published:** 2020-04-14

**Authors:** Nizam Ahmad, El Hassane Anouar, Amalina Mohd Tajuddin, Kalavathy Ramasamy, Bohari M. Yamin, Hadariah Bahron

**Affiliations:** 1 Faculty of Applied Sciences, Universiti Teknologi MARA, Shah Alam, Selangor, Malaysia; 2 Department of Chemistry, College of Science and Humanities in Al-Kharj, Prince Sattam bin Abdulaziz University, Al-Kharj, Saudi Arabia; 3 Atta-ur-Rahman Institute for Natural Product Discovery, Bandar Puncak Alam, Selangor, Malaysia; 4 Faculty of Pharmacy, Universiti Teknologi MARA, Shah Alam, Selangor, Malaysia; 5 Collaborative Drug Discovery Research (CDDR) Group, Pharmaceutical and Life Sciences Community of Research, Universiti Teknologi MARA (UiTM), Shah Alam, Selangor, Malaysia; 6 Publication Enhancement Unit, Penerbit UKM, Universiti Kebangsaan Malaysia, Bangi, Selangor, Malaysia; Aligarh Muslim University, INDIA

## Abstract

This paper reports the synthesis, characterization, anticancer screening and quantum chemical calculation of a tetradentate Schiff base 2,2'-((1*E*,1'*E*)-((2,2-dimethylpropane-1,3-diyl)bis- (azanylylidene))bis(methanylylidene))bis(4-fluorophenol) (**L2F**) and its Pd (II) complex (**PdL2F**). The compounds were characterized *via* UV-Visible, NMR, IR spectroscopy and single crystal x-ray diffraction. Density Functional Theory (DFT) and time-dependent DFT calculations in gas and solvent phases were carried out using B3LYP, B3P86, CAM-B3LYP and PBE0 hybrid functionals combined with LanL2DZ basis set. Complexation of **L2F** to form **PdL2F** was observed to cause a bathochromic shift of the maximum absorption bands of n–π* from 327 to 410 nm; an upfield shift for δ (HC = N) from 8.30 to 7.96 ppm and a decreased wavenumber for ν(C = N) from 1637 to 1616 cm^-1^. Overall, the UV-Vis, NMR and IR spectral data are relatively well reproduced through DFT and TD-DFT methods. **L2F** and **PdL2F** showed IC_50_ of 90.00 and 4.10 μg/mL, respectively, against human colorectal carcinoma (HCT116) cell lines, signifying increased anticancer activity upon complexation with Pd (II).

## Introduction

Schiff bases, also known as imines (C = N) or azomethines (HC = N) were first reported by Hugo (Ugo) Schiff (1834–1915) in 1864 [1]. They are commonly formed through the condensation reaction of primary amines with aldehydes or less commonly, ketones [2]. The resultant imines (R_1_HC = N–R_2_ or R_1_R_2_C = N–R_3_), where R_1_, R_2_ and R_3_ can be any aryl or alkyl groups, can form complexes with metal ions through donation of the lone pair of electrons of nitrogen. One of the most regularly reported *NNOO* ligands are the salen-type ligands, formerly termed for a family of bisimine compounds, N, N′-bis (salicylidine) ethylenediamine derived from salicylaldehyde and ethylenediamine in 2:1 molar ratio [3]. In evolution, the family of the salen-type compounds is not only limited to ligands derived from ethylenediamine but also used to describe ligands derived from other primary diamines such as propanediamine and phenylenediamine. The products of the former are also known as salpn-type ligands and the latter are denoted as salophen-type ligands. This paper focuses on the former type of salen and its corresponding palladium (II) complex.

Schiff base ligands have become progressively popular in coordination chemistry owing to their capability to coordinate with many transition metals, stabilizing them in multiple oxidation numbers. This exceptional chelating ability is mainly empowered by the presence of azomethine nitrogen that carries a lone pair of electrons situated in an sp^2^ hybrid orbital [4]. The effectiveness of Schiff bases as chelating agents is enhanced with the presence of O-H or S-H functional group(s) within 2–3 atomic distances from the azomethine group [5,6]. The stability of Schiff base complexes is largely enhanced through the chelate effect of the polydentate ligands.

Schiff bases are known for their wide potential as bioactive agents such as anticancer [7], antifungal [8,9] and antileishmanial [10]. Previously, we reported the structure-antioxidant activity relationship of a series of phenolic Schiff bases as free radical scavengers using both experimental and DFT calculations [11]. The complexation of Schiff bases as privileged ligands with metals increases their applicability in both chemical and biological processes [12]. Recently, we investigated the bioactivity of an N,O bidentate Schiff base, (E)-(4-methoxybenzylimino)methyl)phenol and its Ni(II) and Pd(II) complexes against HCT116 colorectal cancer cells and Escherichia coli, and the obtained results displayed that the parent ligand is a more superior anticancer and antibacterial agent than positive control [13].

Quantum chemical calculations are considered powerful tools to verify spectral data including the prediction of ^1^H and ^13^C NMR chemical shifts [14–16], UV-Visible absorption bands [17–19] and X-ray structure parameters [20, 21]. Reported studies proved that to predict the excited states of some natural compounds, the use of B3LYP and PBE0 hybrid functionals are appropriate to estimate the excited state energies [22–26]. Previously, we used B3P86 and B3LYP hybrid functionals to predict the maximum absorption bands of a series of natural polyphenols [27]. Lumpi et al. (2013) showed that the M06-2X hybrid functional was suitable to predict the absorption and emission spectra of oligothiophene-based compounds [28]. Quartarolo and Russo applied PBE0 and ab initio multi-reference coupled cluster with the resolution of identity approximation (RICC2) approaches to predict the UV-Visible spectra of pyranoanthocyanins, a class of derived anthocyanin molecules; they showed that the use of larger basis sets results in little improvement of excitation energies, and that the conformational effect has a slight influence on the λMAX predictions [29]. In another study, Sousa et al. (2012) tested B3LYP and PBE0, and long-range corrected ωB97X and ωB97XD hybrid functional to predict the absorption electronic spectra of the isopentaphyrin derivative and its lutetium complex; and they showed that ωB97XD is the most reliable to reproduce the absorption electronic spectra of the isopentaphyrin derivative and its lutetium complex [30]. In regard to the ^1^H and ^13^C chemical shift calculations, the gauge-independent atomic orbital (GIAO) method is one of the most common approaches used to predict nuclear magnetic shielding tensors (σiso) [31, 32].

The present study aims at investigating anticancer activity of a synthesized Schiff base L2F and its palladium (II) complex PdL2F. The ligand and its complex were characterized by single crystal x-ray diffraction, NMR, IR and UV-Vis spectroscopic techniques. To support the experimental data, DFT and TD-DFT calculations in gas and solvent phases were carried out to predict maximum absorption bands, vibrations modes and chemical shifts of L2F and PdL2F. In addition, the chelation effect of L2F on the experimental data is emphasized.

## Material and methods

### Starting materials and instruments

All chemicals and solvents purchased from commercial suppliers were used without further purification. The elemental analysis (C, H, and N) of ligand, L2F and complex, PdL2F were obtained from Thermo Scientific Flash 2000 Elemental Analyser. Melting points were determined using Stuart SMP10. Perkin-Elmer Spectrum One FTIR spectrometer using KBr pellets recorded infrared spectra of ligand, L2F and complex, PdL2F between 450–4000 cm-^1^. ^1^H and ^13^C NMR spectra were recorded on a Bruker Varian 600 MHz spectrometer as CDCl_3_ or DMSO-d6 solutions.

X-ray single crystal data for the coordination polymers were collected at 293(2) and 171(1) K on Bruker D8 QUEST with photon CCD area-detector diffractometer Mo-Kα (λ = 0.71073 Å). High-quality crystals were chosen using a polarizing microscope and mounted on a glass fibre. Data processing and absorption correction was performed using a multi-scan method. The structures were solved by direct method using SHELXS. All data were refined by full matrix least-squares refinement against |F2| using SHELXL [33], and the final refinement include atomic position for all the atoms, anisotropic thermal parameters for all the non-hydrogen atoms, and isotropic thermal parameters for the hydrogen atoms. The programs PLATON and Mercury were used throughout the study [34].

### Synthesis

#### Synthesis of Schiff base L2F

**Fig 1:**
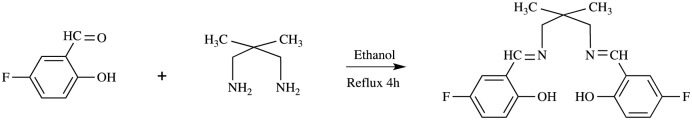
Synthesis of L2F.

**L2F** was prepared by adding a 10-mL hot ethanolic solution of DMPD (1 mmol, 0.1022 g) into a stirred equivolume ethanolic solution of 5-fluorosalicylaldehyde (2 mmol, 0.2802 g) (Fig 1). The solution was refluxed over a period of 4 h, cooled to room temperature and chilled overnight. The yellow precipitate obtained was filtered off, washed with cold EtOH and air-dried. The yellow single crystal of the compound was obtained upon slow evaporation from ethanol. The synthesized compound was identified as 2,2'-((1*E*,1'*E*)-((2,2-dimethylpropane-1,3-diyl)bis(azanylylidene))bis(methanylylidene))bis(4-fluorophenol), a tetradentate Schiff base assigned as L2F. Yellow solid; yield, 85.0%; m.p. 115–116 °C. Elemental analysis for L2F, analysed as C_19_H_20_F_2_N_2_O_2_ (326.28 gmol^-1^); % Found (Calc.) C, 65.81 (65.88): H, 5.80 (5.82); N, 8.23 (8.09); UV-Vis bands (MeCN, nm): π-π* (C = N) 257, n-π* (C = N) 327; IR bands (KBr pellet, cm^-1^): ν(O-H) 3271, ν(C = N) 1637, ν(C-O) 1071, ν(C = C) 1583, ν(C-H sp^2^) 3071, ν(C-H sp^3^) 2956; ^1^H NMR (500 MHz, CDCl_3_, ppm): δ(OH) 13.25 (s, 1H), δ(HC = N) 8.30 (s, 1H); ^13^C NMR (500 MHz, CDCl_3_, ppm): δ(C-OH) 157.74, δ(C = N) 164.74.

#### Synthesis of complex PdL2F

**Fig 2:**
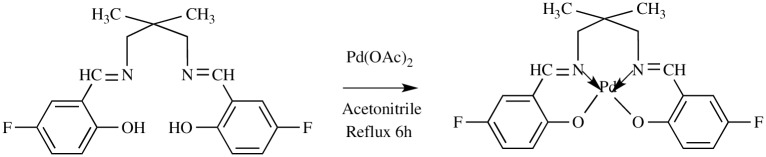
Synthesis of PdL2F.

**PdL2F** was synthesized by dissolving palladium (II) acetate (1 mmol, 0.2248 g) in 20 mL of acetonitrile in a round bottom flask.1mmol, 0.3464 g of **L2F** was dissolved separately in 20 mL of acetonitrile (Fig 2). The ligand solution was added dropwise into the flask containing the metal salt solution with stirring, then refluxed for 6 h. The dark yellow precipitate was filtered off, washed with a small amount of cold acetonitrile and air-dried. The yellow single crystal of the complex was obtained upon slow evaporation of DMSO: MeOH (1:1 v/v). The synthesized compound was identified as 2,2'-((1*E*,1'*E*)-((2,2-dimethylpropane-1,3-diyl)bis-(azanylylidene))bis(methanylylidene))bis(4-fluorophenol)palladium(II), a tetradentate Schiff base complex denoted as PdL2F. Yellow solid; yield, 55.5%; m.p. 295–300 °C. Elemental analysis for PdL2F, analysed as C_19_H_18_F_2_N_2_O_2_Pd (450.78 gmol^-1^); % Found (Calc.) C, 50.10 (50.63): H, 3.92 (4.03); N, 6.92 (6.21); UV-Vis bands (MeCN, nm): π-π* (C = N) 255, n-π* (C = N) 410; IR bands (KBr pellet, cm^-1^): ν(C = N) 1616, ν(C-O) 1079, ν(C = C) 1545, ν(C-H sp^2^) 3041, ν(C-H sp^3^) 2960; ^1^H NMR (500 MHz, CDCl_3_, ppm): δ(HC = N) 7.96 (s, 1H); ^13^C NMR (500 MHz, CDCl_3_, ppm): δ(C = N) 164.40.

#### Cytotoxicity test

The HCT 116 (ATCC, CCL247) colorectal carcinoma cells were obtained from the American Type Culture Collection (ATCC), Manassas, VA, USA. The cells were maintained in Roswell Park Memorial Institute (RPMI) 1640 medium supplemented with 10% heat activated fetal bovine serum and 1% penicillin-streptomycin at 37°C in a 5% CO_2_ atmosphere. The HCT116 cells were seeded at 2,000 cells/ 200 μL onto each well of the 96-well plate and were treated with Schiff base ligand (L2F) and palladium(II) (PdL2F) (0.01–100 μg/mL) followed by incubation for 72 hours at 37°C and 5% CO_2_ overnight. DMSO (0.001–10%) and 5-FU (0.01–100 μg/mL) were also included as vehicle and positive controls, respectively. Treated cells were fixed with 10% (w/v) tricholoracetic acid (TCA) at 4 °C for 30 minutes. The fixed cells were washed with tap water. Each well was stained with 0.4% (w/v) SRB solution at room temperature for 10–15 minutes. The plate was rinsed with (1% v/v) acetic acid for the removal of the unbound dye and left to dry overnight. Tris base (10 mM) was added for dye solubilisation. The plate was read at 570 nm. Data obtained was used to plot the dose-response curve from which IC_50_ was determined. IC_50_ value is defined as the concentration of a test compound required to achieve half maximal inhibition [35].

### Theoretical details

Geometry optimisation and frequency calculations of **L2F** Schiff base and its corresponding complex with palladium metal were performed using DFT method. Five hybrid functionals B3LYP, B3P86, CAM-B3LYP and PBE0 combined with LanL2DZ basis set were tested [36]. The true minima of the optimized structure were confirmed by the absence of imaginary frequencies. The calculated vibrational modes were scaled by a factor of 0.9679 [37]. Excited states (ES) calculations were performed using TD-DFT method. The maximum absorption bands, vertical electronic excitations and oscillator strengths (*f*>0 for allowed transition) were calculated using the five hybrid functions [38, 39]. The predicted magnetic isotropic shielding tensors (σ) were calculated using the standard Gauge-Independent Atomic Orbital approach (GIAO) [40], using the hybrid functional B3LYP combined with LanL2DZ basis set. The isotropic shielding values were used to calculate the isotropic chemical shifts δ with respect to the reference tetramethylsilane (Si(CH_3_)_4_). δ_iso_(X) = σ_TMS_(X)–σ_iso_(X), where δ_iso_ is isotropic chemical shift and σ_iso_ isotropic shielding constant. The predicted chemical shifts were obtained using the equation δ_exp_ = aδ_cal_+b, where δ_cal_ = δ_iso_. The solvation effect was considered implicitly using polarisable continuum model (PCM) [41]. In such model, the tilted substrates were embedded into a shape-adapted cavity surrounded by a dielectric continuum solvent, described by its dielectric constant (e.g., ε_CDCl3_ = 4.7113). The PCM has been reported to correctly model major solvent effects such as electrostatic effects of the medium, providing no specific solute-solvent interactions such as hydrogen bond interactions, dipole-dipole interactions, or induced dipole-dipole interactions [42]. For excited state calculations, the solvent effects were considered by using IEF-PCM and state-specific solvation (SS-PCM) [43, 44]. DFT calculations were performed using Gaussian09 package [45].

## Results and discussion

### UV-visible spectroscopy

**Fig 3:**
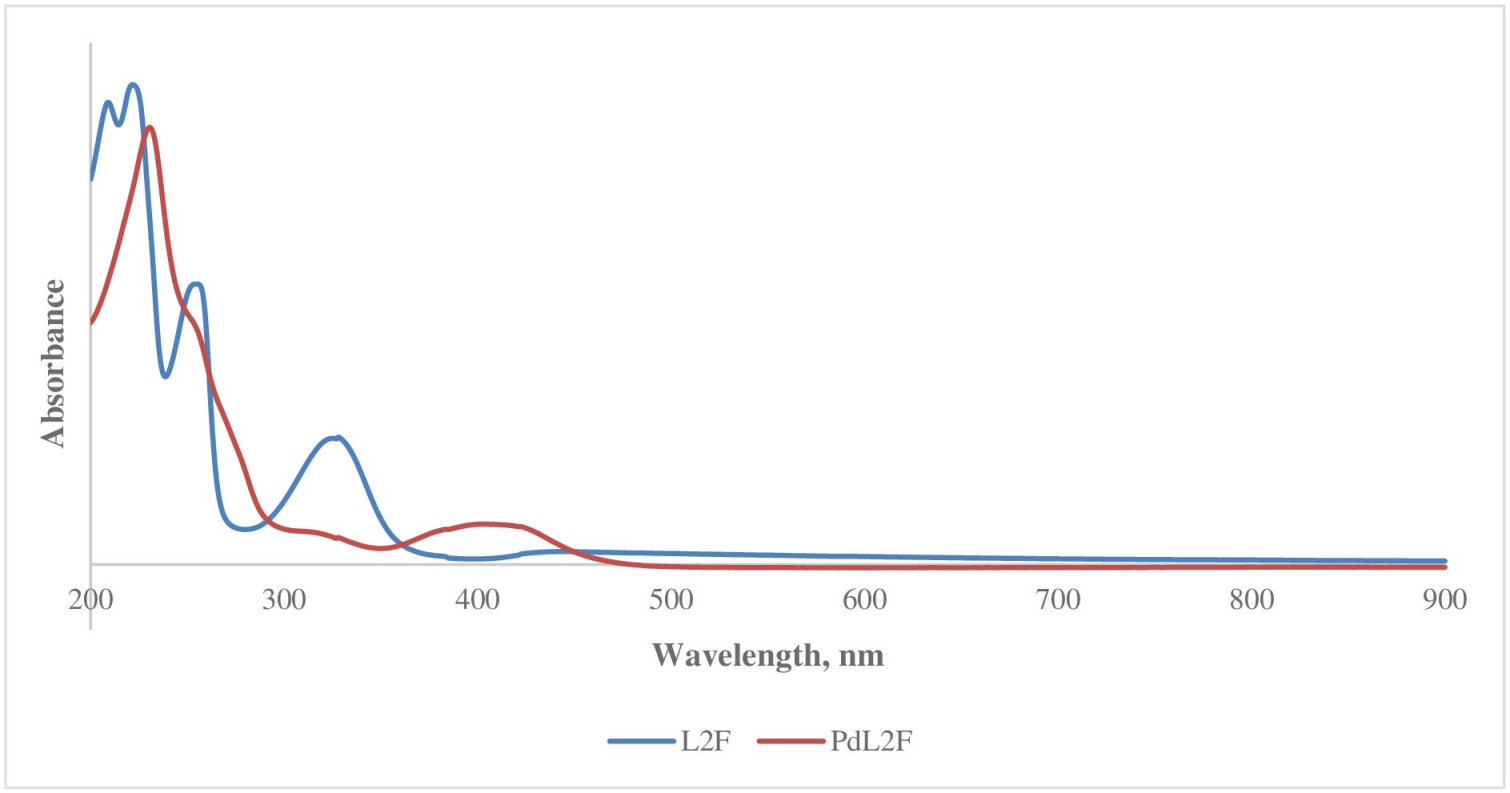
UV-vis spectra of L2F and PdL2Fs.

The main experimental and predicted maximum absorption bands of **L2F** Schiff base and **PdL2F** are shown in Tables 1 and 2. The observed λ_MAX_ at 257 nm as shown in Fig 3 is attributed to π-π* electronic transition of the C = N chromophore, and it is in accordance with the value reported by Khanmohammadi, Salehifard, & Abnosi, (2009) [46]. Theoretically, this band corresponds to an electronic transition between HOMO and LUMO orbitals. As can be seen from Table 3, this band was well reproduced with the B3LYP and B3P86 hybrid functionals in gas, IEF-PCM and SS-PCM phases (Table 1). For instance, by using B3LYP hybrid functional for **L2F**, variations of 2, 0 and 3 nm with respect to the experimental value were obtained in gas, IEF-PCM and SS-PCM phases, respectively. This band was slightly influenced by the solvatochroism effect where both models IEF-PCM and SS-PCM showed negligible shifts of the corresponding value obtained in gas phase with both B3LYP and B3P86 hybrid functional with variation less than 3 nm. The solvent effect may arise from intermolecular H-bonding or weaker van der Waal’s forces between the F and O-containing Schiff base with the solvent, acetonitrile. CAM-B3LYP and PBE0 failed in the reproduction of the experimental value of the observed band at 275 nm. CAM-B3LYP and PBE0 hybrid functionals for **L2F** underestimated the experimental value with variations of 22 and 10 nm in gas phase, respectively. The complexation of the Schiff base ligand **L2F** with Pd (II) has an effect on the observed band at 257 nm with slight hypsochromic shift (blue shift) of 2 nm. Similarly, the band at 255 nm of **PdL2F** complex was well reproduced with B3P86 and B3LYP, while it is underestimated with CAM-B3LYP and PBE0 hybrid functionals with variations of 32 and 8 nm, respectively.

**Table 1:** λ_MAX_ (nm), E_MAX_ (eV), *f* of π→π* transitions of L2F and PdL2F calculated using B3LYP, PBE0, CAM-B3LYP and PBE0 hybrid functional in gas, IEF-PCM and SS-PCM.

	Gas			IEF-PCM			SS-PCM			Experimental	
	λ_MAX_	E_MAX_	*F*	λ_MAX_	E_MAX_	*f*	λ_MAX_	E_MAX_	*f*	λ_MAX_	E_MAX_
**B3LYP**											
L2F	259	4.79	0.29	257	4.82	0.48	260	4.77	0.67	257	4.82
PdL2F	259	4.78	0.20	258	4.80	0.39	261	4.75	0.60	255	4.86
**B3P86**											
L2F	258	4.81	0.28	256	4.84	0.47	259	4.79	0.67	257	4.82
PdL2F	256	4.84	0.16	254	4.87	0.32	257	4.83	0.48	255	4.86
**CAM-B3LYP**											
L2F	235	5.28	0.38	234	5.29	0.46	237	5.23	0.62	257	4.82
PdL2F	223	5.57	0.68	232	5.35	0.65	236	5.25	0.93	255	4.86
**PBE0**											
L2F	247	5.01	0.47	247	5.02	0.58	250	4.95	0.77	257	4.82
PdL2F	247	5.02	0.25	245	5.05	0.48	248	4.99	0.68	255	4.86

**Table 2:** λ_MAX_ (nm), E_MAX_ (eV), *f* of electronic transitions of L2F (n→π*) and its complexes and PdL2F (n→d) calculated using B3LYP, PBE0, CAM-B3LYP and PBE0 hybrid functional in gas, IEF-PCM and SS-PCM.

	Gas			IEF-PCM			SS-PCM			Experimental	
	_λMAX_	_EMAX_	_f_	_λMAX_	_EMAX_	_f_	_λMAX_	E_MAX_	*f*	_λMAX_	E_MAX_
**B3LYP**											
L2F	337	3.68	0.14	327	3.80	0.18	332	3.73	0.23	327	3.79
PdL2F	366	3.38	0.08	352	3.52	0.08	356	3.48	0.13	410	3.02
**B3P86**											
L2F	339	3.66	0.13	331	3.75	0.18	336	3.69	0.22	327	3.79
PdL2F	362	3.43	0.08	407	3.05	0.06	411	3.02	0.12	410	3.02
**CAM-B3LYP**											
L2F	301	4.12	0.20	293	4.23	0.22	299	4.15	0.27	327	3.79
PdL2F	365	3.40	0.16	346	3.59	0.21	353	3.51	0.33	410	3.02
**PBE0**											
L2F	325	3.81	0.17	318	3.90	0.19	324	3.83	0.24	327	3.79
PdL2F	347	3.57	0.08	387	3.20	0.09	392	3.16	0.17	410	3.02

**Table 3:** Calculated, scaled and experimental vibrational modes of L2F and its complexes PdL2F.

	L2F			PdL2F		
	Cal	Scal	Exp	Cal	Scal	Exp
**ν**_**O-H**_						
B3LYP	2349	2274	2393	-	-	-
B3P86	2092	2025	2393	-	-	-
CAM-B3LYP	2506	2426	2393	-	-	-
PBE0	2214	2143	2393	-	-	-
**ν**_**C-H**_ **(ar)**						
B3LYP	3250	3146	3274	3245	3141	3225
B3P86	3269	3164	3274	3265	3160	3225
CAM-B3LYP	3277	3172	3274	3276	3171	3225
PBE0	3280	3175	3274	3276	3171	3225
**ν**_**C = N**_						
B3LYP	1661	1608	1637	1643	1590	1616
B3P86	1667	1613	1637	1662	1609	1616
CAM-B3LYP	1712	1657	1637	1695	1641	1616
PBE0	1688	1634	1637	1677	1623	1616
**ν**_**C-H(sp**_^**2**^_**)**_						
B3LYP	3108	3008	3071	3139	3038	3041
B3P86	3133	3032	3071	3155	3054	3041
CAM-B3LYP	3138	3037	3071	3170	3068	3041
PBE0	3141	3040	3071	3251	3147	3041
**ν**_**C-H(sp**_^**3**^_**)**_						
B3LYP	3057	2959	2956	3037	2940	2960
B3P86	3044	2946	2956	3051	2953	2960
CAM-B3LYP	3049	2951	2956	3061	2963	2960
PBE0	3059	2961	2956	3061	2963	2960

Cal = Calculated; Scal = Scaled; Exp = Experimental

The strong maximum absorption band observed at the higher wavelength of 327 nm in the UV-Vis spectrum of **L2F** is assigned to n-π* electronic transition of the imine chromophore. This band indicates that there is a transition of electrons from the non-bonding n orbital to the anti-bonding π* orbital [47, 48]. Theoretically, this band corresponds to an electronic transition between HOMO and LUMO orbitals of **L2F** Schiff base. Referring to Table 2, in the gas phase, λ_MAX_ at 327 nm was overestimated with the B3LYP and B3P86 functionals and underestimated with CAM-B3LYP and PBE0 functionals. The best reproduction for the gas phase was obtained with the PBE0 hybrid functional with a variation of 2 nm with respect to the experimental value. This λ_MAX_ was seen to be strongly affected by the solvent with hypsochromic shifts (blue shift) obtained in different hybrid functionals. The exact reproduction of λ_MAX_ of **L2F** at 327 in IEF-PCM was obtained with B3LYP hybrid functional, while in SS-PCM the best reproduction was obtained with PBE0 hybrid functional with variation of 3 nm. The blue shift of λ_MAX_ at 327 nm in solvent is mainly referred to the solute-solvent interactions and to the formation of hydrogen bonding of L2F with solvent molecules. The results are in agreement with the values and trend reported by More et al., (2017) [49].

Upon complexation with Pd (II), the λ_MAX_ experienced a bathochromic (red) shift to a higher wavelength of 410 nm. This is in agreement with the weakening of the C = N when the lone pair of electrons on N is donated to the Pd (II) in a Lewis acid-base interaction. The best reproduction of this band was obtained with B3P86 in SS-PCM and IEF-PCM with variation of 1 and 3 nm with respect to the experimental value, respectively. It is worth to mention that the solute-solvent interactions induce a bathochromic shift (red shift) of the n-π* band.

### Infrared spectroscopy

**Fig 4:**
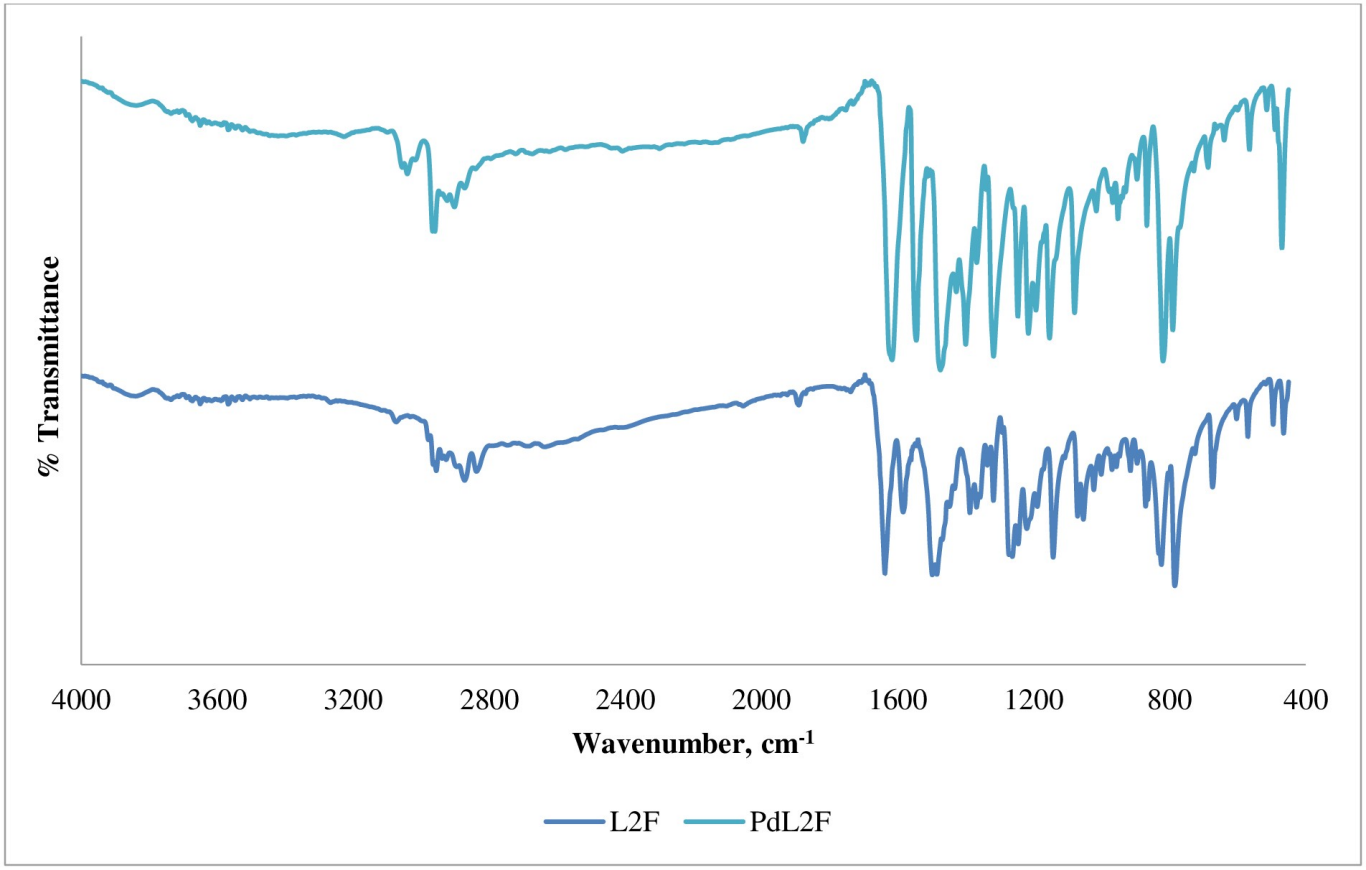
FTIR spectra of L2F and PdL2F.

The calculated, scaled and experimental main vibrational modes of **L2F** and **PdL2F** are reported in Table 3. The characteristic peak for imine, ν(C = N), is found at 1637 cm^-1^ in the spectrum of the free ligand, L2F. The best reproduction of this band was obtained with PBE0 hybrid functional with a variation of 3 cm^-1^ with respect to the observed value (Table 3). Other tested functionals namely B3LYP, B3P86 and CAM-B3LYP, reproduced the peak less accurately with variations of 31, 24 and 20 cm^-1^ with respect to the observed value, respectively. This vibration mode experienced a shift of 21 cm^-1^ to a lower frequency of 1616 cm^-1^ in the spectrum of PdL2F as can be seen in Fig 4, indicating that complexation has been established through bonding of imine nitrogen and Pd (II) center [50]. The C = N bond became weaker upon complexation as a result of the inductive effect of lone electron pair on imine nitrogen being shared with the metal centre.

The weak vibration of hydroxyl group ν(OH) of **L2F** is found at 2393 cm^-1^. The best reproduction of this vibration mode was obtained with B3LYP functional with a variation of 44 cm^-1^, while other tested functionals failed in its reproduction with variation higher than that. The weak peak of OH indicated the occurrence of hydrogen bonding OH···N = C occurring in intramolecular manner between –OH with imine nitrogen [51], as reported in Table 7. Noticeably, this signal disappeared in the PdL2F complex signalling that the complexation was established through deprotonation of the phenolic hydroxyl [52] prior to bonding with Pd (II) center. The shifting of ν(C-O) peak to a higher frequency by 8 cm^-1^ in PdL2F complex further supported the involvement of phenolic oxygen in complexation [53, 54].

### NMR spectroscopy

**Fig 5:**
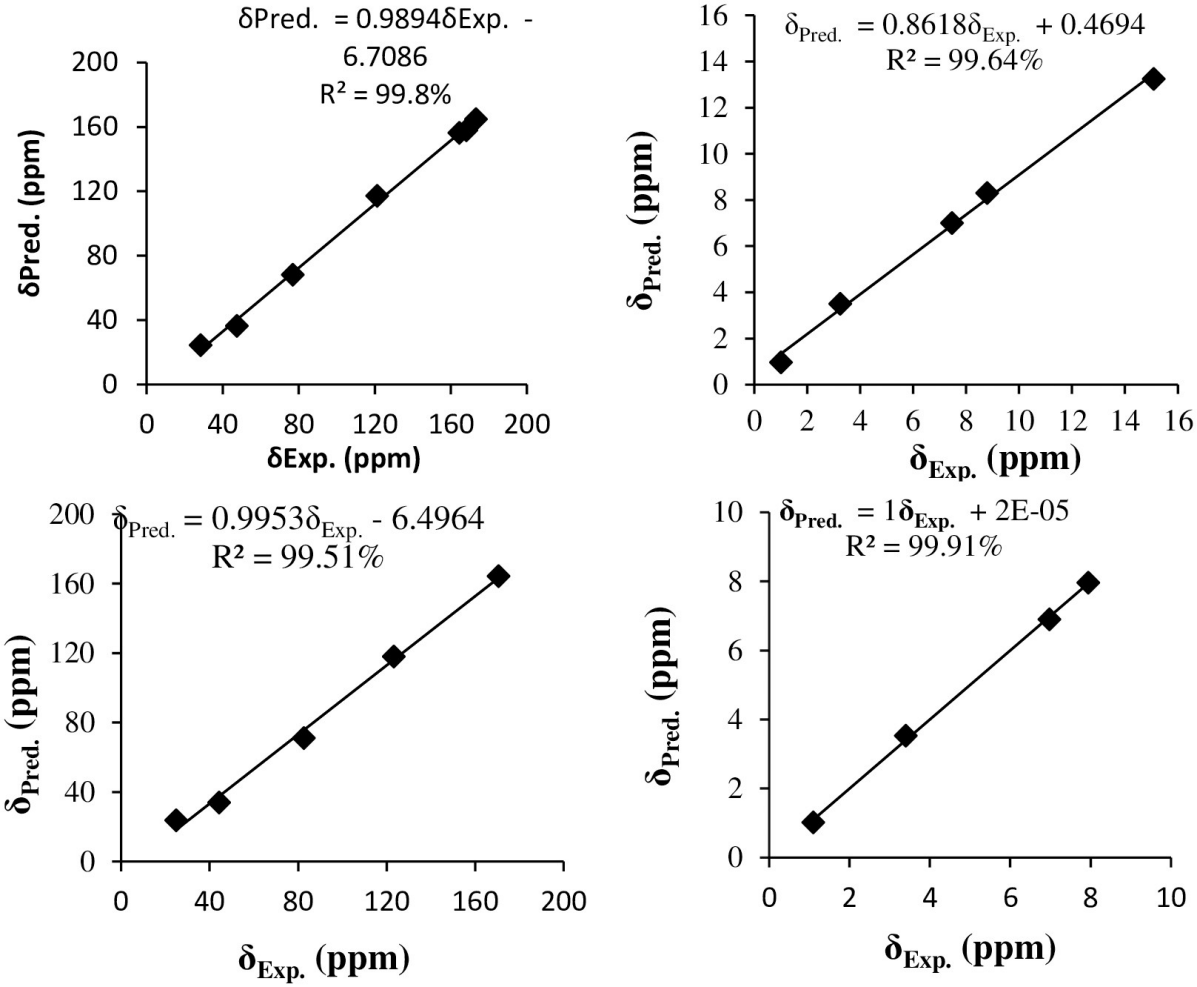
Correlation curves between the experimental and predicted ^13^C NMR (right) and ^1^H NMR (left) chemical shifts of L2F Schiff base (up) and its complex PdL2F (bottom).

The experimental and the predicted ^1^H and ^13^C chemical shifts of **L2F** and **PdL2F** are shown in Table 4 and Fig 5. The observed proton of OH group of **L2F** appears as singlet in the downfield region at 13.25 ppm due to the formation of intramolecular hydrogen bonding with the imine nitrogen [50]. Theoretically, this chemical shift was well reproduced in in gas and PCM phases, with variations of 0.45 and 0.25 ppm, respectively. The absence of OH peak in the spectra of PdL2F supported the infrared evidence that the coordination to metal centres was established through deprotonation of the hydroxyl groups [55]. The chemical shift for the imine proton, HC = N, was detected as a singlet in the region of 8.32 ppm for **L2F**. The predicted corresponding one in gas and PCM phases appeared at 7.9 and 8.0 ppm, respectively. The complexation of the ligand with Pd (II) shifted this peak downfield to 7.96 ppm. This shift was reproduced theoretically, appearing at 7.9 ppm. This supports the observation made in IR spectroscopy of the involvement of imine nitrogen in the coordination to the metal centre [56].

**Table 4:** Predicted and experimental ^1^H and ^13^C chemical shifts of L2F and its complex PdL2F.

	L2F			PdL2F		
^1^H-NMR	Gas	PCM	Exp.	Gas	PCM	Exp.
OH	12.8	13.5	13.25			-
HC = N	7.9	8.0	8.30	7.9	7.9	7.96
Ar-H	6.8	6.9	6.91–7.35	7.0	7.0	6.76–7.20
CH_3_	1.5	1.3	0.97	1.2	1.1	1.02
CH_2_	3.1	3.3	3.5	3.3	3.4	3.53
^**13**^**C-NMR**						
C (OH)	160	167	157.74	-		ND
HC = N	164	172	164.74	164	163	164.4
Ar-C	113	121	116.35–119.51	115	116	117.76–120.66
Ar-F	157	164	156.34	-		ND
CH_3_	21	29	24.36	18	18	23.66
-CH_2_-	70	77	68.2	76	76	71.02
-C(CH_3_)_2_	40	48	36.3	37	38	33.95

The experimental and predicted chemical shifts of aromatic protons of free ligands appeared as multiplets in the range of 6.78–7.19 ppm for the free ligand. This is in agreement with the chemical shifts reported by Ahmad et al., (2018) [57]. These hydrogens experience the shielding effect of diamagnetic anisotropy caused by circulating π electrons in the aromatic rings [58]. The signals of these aromatic protons were found at 6.77–7.18 ppm in the complex. The shifting in chemical shift of these protons indicates that the complexation has been established between metal ions and the ligands. Good correlations are obtained between the experimental and the predicted proton chemical shifts of **L2F** and its complex **PdL2F** with correlation coefficient coefficients of 99.64 and 99.91%, respectively.

The ^13^C NMR chemical shift of azomethine carbon, HC = N is found at 164.74 ppm in the free ligand. The corresponding predict one appears at 164 ppm in gas phase. However, in PCM phase downfield of the signal is observed with a variation of 7.26 ppm with respect to the experimental value. The signal of C-OH is observed at 158 ppm, slightly up field with respect to HC = N signal. Characteristic peaks of aliphatic and aromatic methyl were observed at 36.30 ppm and 24.36 ppm, respectively. Aromatic carbons were detected in the range of 117.96–156.34 ppm. The ^13^C NMR chemical shifts of **L2F** and **PdL2F** are well reproduced with correlation coefficients of 99.8 and 99.51%, respectively (Fig 5).

### Crystal structure description

The ligand **L2F** and complex **PdL2F** crystallized in Pī and monoclinic system respectively. The crystal system and refinement parameters are given in Table 5.

**Table 5:** Crystallographic data and refinement parameters for L2F and PdL2F.

Compound	L2F	PdL2F
**Chemical formula**	C_19_H_20_F_2_N_2_O_2_	C_19_H_18_F_2_N_2_O_2_Pd
**Molecular weight**	346.37	450.75
**Temperature (K)**	0(2)	273(2)
**Wavelength (Å)**	0.71073	0.71073
**Crystal system**	Triclinic	monoclinic
**Space group**	Pī	P21/c
**Unit cell dimensions (Å)**	a = 6.1334(5), b = 9.2490(7), c = 16.3270(13)	a = 11.111(2), b = 13.701(3), c = 12.196(2)
**Angles(°)**	α = 102.110(2), β = 96.032(2), γ = 103.520(2)	β = 105.069(6)
**Volume (Å**^**3**^**)**	868.84(12)	1792.8(6)
**Z**	2	4
**Absorption coefficient (mm**^**-1**^**)**	0.101	1.313
**F(0 0 0)**	364	904
**Crystal size**	0.50 × 0.29 × 0.19 mm	0.27 × 0.20 × 0.08 mm
**No. of reflections collected**	25298	51046
**No. of independent reflections**	4327	4443
***θ* range (°)**	2.930–28.370	2.973–28.353
**Index ranges**	-7< = h< = 8	-14< = h< = 14
	-12< = k< = 12	-18< = k< = 18
	-21< = l< = 21	-16< = l< = 16
**Goodness-of-fit on F2**	1.052	1.106
***R* indices**	*R1* = 0.0626, *wR2* = 0.1294	*R*1 *=* 0.0646, *wR*2 *=* 0.1229
***R* indices (all data)**	*R1* = 0.1003, *wR2* = 0.1500	*R*1 *=* 0.0925, *wR*2 *=* 0.1428
**CCDC no.**	1855625	1890435

**Fig 6:**
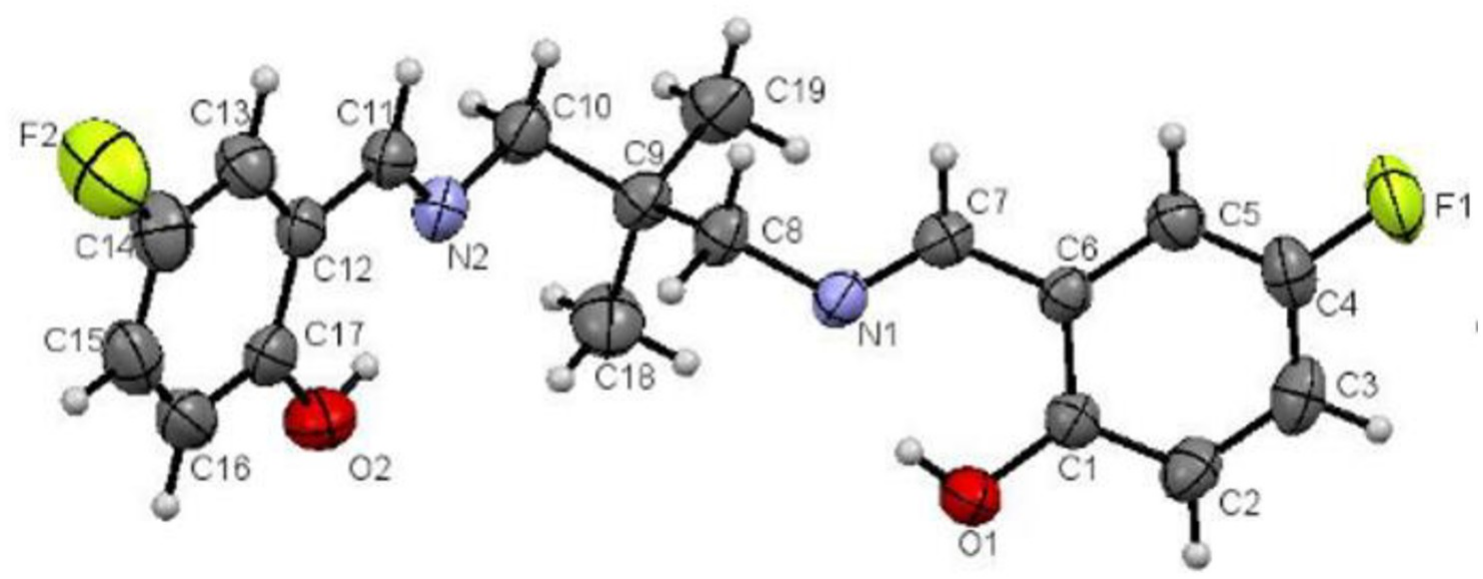
Molecular structure of ligand drawn at 50% probability ellipsoids.

Fig 6 shows the molecular structure of the ligand with the numbering scheme. The molecule consists of two 2-fluoro (2-iminoethyl) phenol groups linked by 2,2-dimethyl-1,3- dimethylenepropane moiety.

The two 2-fluoro (2-iminoethyl) phenol groups F1/O1/N1/C1-C8 and F2/O2/N1/C10-C17 are each planar with maximum deviation of 0.039(2) and 0.048(2) Å for fluorine atom (F1 and F2) respectively from their mean square planes. Both planes make dihedral angle of 42.26°. The N1/C8/C9 and N2/C10/C9 are planar with maximum deviation of 0.008 (2) Å for atom N1 from the least square plane and the torsion angle of C9-C8-N1-C7 and C9-C10-N2-C11 are 147.68 ° and 138.19(19) ° respectively. The bond length and angles are in normal ranges (Table 6). There are two intramolecular hydrogen bonds O1-H1A…N1 and O2-H2A…N2 in the molecule (Table 7). No intermolecular hydrogen bonds were observed.

**Table 6:** Some important bond lengths (Å) and angles (°) of L2F and PdL2F.

Bond length/angle	L2F	PdL2F
**O1-C1**	1.348(2)	1.306(7)
**O2-C17**	1.349(3)	1.308(7)
**N1-C7**	1.270(2)	1.280(8)
**N1-C8**	1.458(2)	1.480(7)
**N2-C11**	1.270(3)	1.297(8)
**N2-C10**	1.456(3)	1.467(7)
**Pd1-O1**	-	1.994(4)
**Pd1-N2**	-	1.997(5)
**Pd1-O2**	-	2.000(4)
**Pd1-N1**	-	2.013(4)
**O1-Pd1-N2**	-	172.85(18)
**O1-Pd1-O2**	-	80.42(17)
**N2-Pd1-O2**	-	92.49(18)
**O1-Pd1-N1**	-	92.33(18)
**N2-Pd1-N1**	-	94.79(19)
**O2-Pd1-N1**	-	172.33(18)
**C8-C9-C10-N2**	-	-73.8(7)
**C19-C9-C10-N2**	-	170.5(5)
**C18-C9-C10-N2**	-	50.4(7)

**Table 7:** Hydrogen bonds in the ligand L2F and PdL2F complex.

D --H ..A	D --H (Å)	H ..A (Å)	D --A (Å)	D --H ..A (°)
**L2F**				
**O1 --H1B ..N1**	0.83(2)	1.86(3)	2.619(2)	152(3)
**O2 --H2B ..N2**	0.82(3)	1.84(4)	2.599(3)	153(3)
**PdL2F**				
**C5 --H5A ..O1**	0.93	2.46	3.376(8)	169

(symmetry codes; i = x,1/2-y,1/2+z)

**Fig 7:**
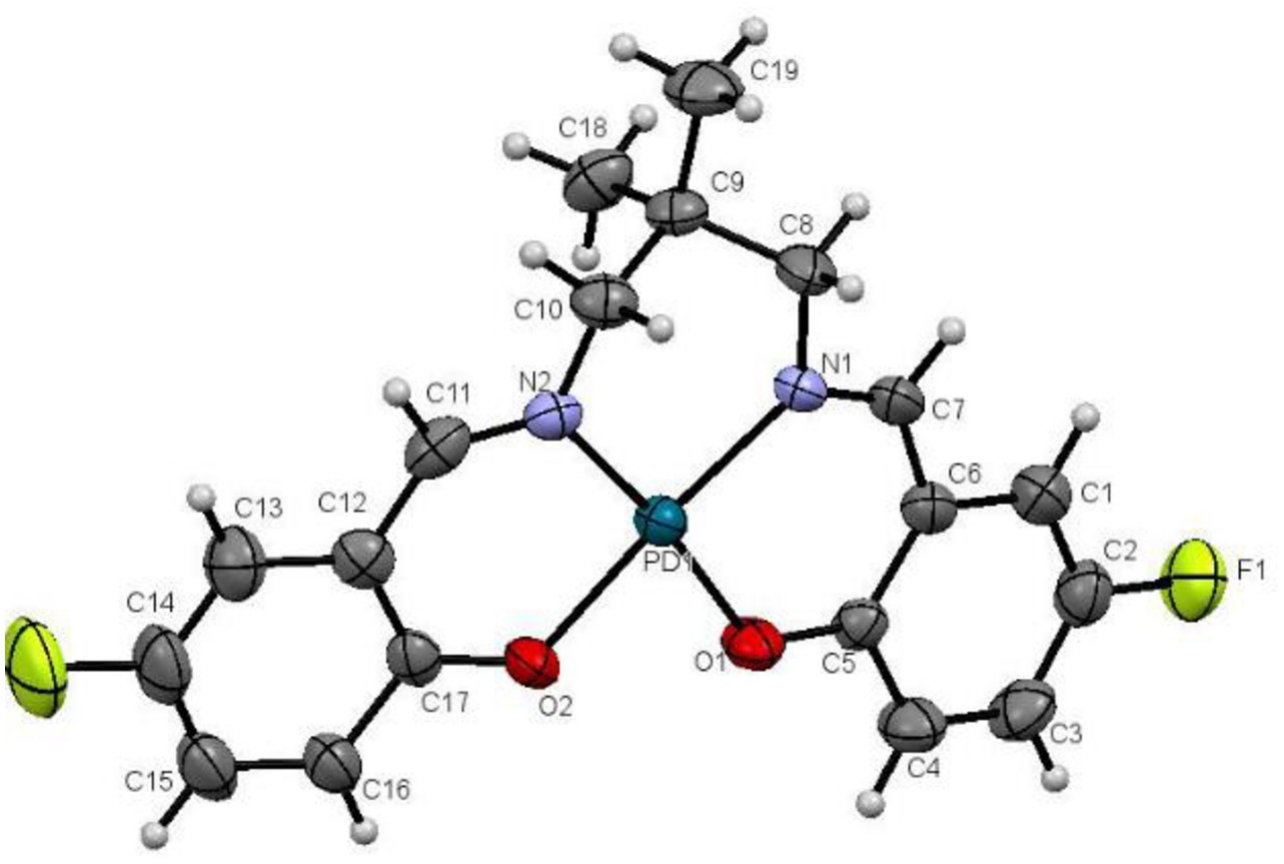
Molecular structure of PdL2F complex drawn at 50% probability ellipsoids.

The ligand formed 1:1 complex with palladium acetate through coordination via nitrogen and phenolic oxygen atoms to form a square planar geometry (Fig 7). Both phenolic OH groups were deprotonated. The nitrogen atoms are at *trans* position to oxygen atoms. The *trans* N-Pd1-O angles are about 172° and the cis angles about the central Pd1 atom between 80.42 (17) and 94.79 (19)° indicate a distorted square planar geometry.

The coordination also creates three 6-membered rings, Pd1-N1-C7-C8-C5-O1, Pd1-N2-C11-C12-C17-O2 and Pd1-N1-C8-C9-C10-N2. The first two rings are essentially planar with maximum deviation of 0.079 (4) Å for O2 atom from the least square plane. However, the Pd1-N1-C8-C9-C10-N2 ring adopts half chair conformation with C9 atom deviated by 0.404(6) Å for C10 atom from the least square plane (Fig 7).

The O1-C1 and O2-C17 bond lengths in the complex are significantly shorter than that in the ligand as the result of the deprotonation of the phenolic groups. On the other hand, the N1-C7 and N2-C11 bond lengths are slightly longer than that in the ligand. Other bond lengths and angles are in normal ranges and comparable with those reported by Ahmad et al., (2017) [59].

**Fig 8:**
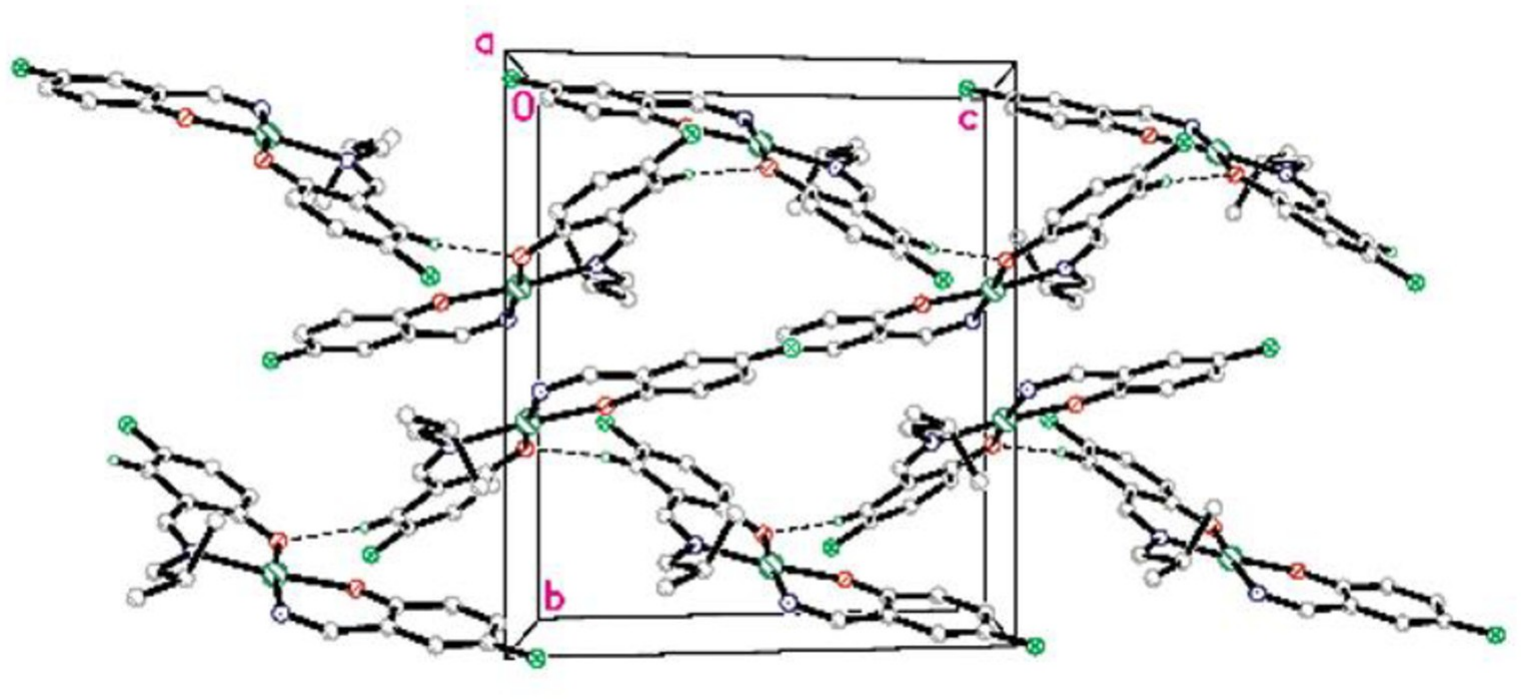
Molecular packing of PdL2F complex viewed down a axis. The non-hydrogenbonded hydrogen atoms are omitted for clarity.

In the crystal structure the complex is stabilized by C5-H5A…O1 intermolecular hydrogen bonds (symmetry code as in Table 7) to a zig-zag one dimensional chain along the c axis (Fig 8).

### Biological activity screening

**L2F** and **PdL2F** were screened for their anticancer properties against HCT116. As depicted in Table 8, **PdL2F** showed a better potency against the cancer cell with IC_50_ of 4.1 μg/mL while **L2F** could be considered less active as anticancer agent with 90.00 μg/mL. Located in the same group as platinum, as well as being a soft metal with an abundance of *d* electrons, palladium is expected to interact with the DNA backbone that is rich with N, a soft donor atom. The excellent performance of the complex may also be caused by its square planar geometry which allowed them to stack in the structure of the DNA [60]. The structure helped palladium (II) complex kinking the pharmacological target, DNA, more effectively through a mechanism called intercalation [61]. Intercalation has been proven to stabilize, lengthen, stiffen, and at certain extent unwinds the DNA double helix. Intercalators can fit between two base pairs, opening a space between its base pairs by unwinding [62].

**Table 8:** The anticancer activity of L2F and its PdL2F complex against HCT116.

Compound	IC_50_, μg/mL
**L2F**	90.00 (less active)
**PdL2F**	4.1 (active)
**Positive control (5-FU)**	1.70
Vehicle Control (DMSO)*	1.09

### Conclusion

2,2'-((1E,1'E)-((2,2-dimethylpropane-1,3-diyl)-bis(azanylylidene))bis(methanylylidene))bis(4-fluorophenol), a tetradentate Schiff base named **L2F** and its complex **PdL2F**, were successfully synthesised. The molecular structures of the synthesised compounds were confirmed by spectroscopic and X-ray techniques. Anticancer activity of **PdLF** was better than its parent ligand against HCT1116 with 1C_50_ of 4.1 μg/mL.

## Supporting information

S1 Fig ^1^H NMR spectra of L2F and PdL2F. (DOCX)

S2 Fig ^13^C NMR spectra of L2F. (DOCX)

S1 Table Crystallographic information of L2F. (CIF)

S2 Table Crystallographic information L2F. (CIF)
