# Correction: Synthesis, characterization, quantum chemical calculations and anticancer activity of a Schiff base *NNOO *chelate ligand and Pd(II) complex

**DOI:** 10.1371/journal.pone.0249437

**Published:** 2021-03-25

**Authors:** The PLOS ONE staff

The first author’s name is spelled incorrectly. The correct name is: Shahrul Nizam Ahmad. The correct citation is: Ahmad SN, Anouar EH, Tajuddin AM, Ramasamy K, Yamin BM, Bahron H (2020) Synthesis, characterization, quantum chemical calculations and anticancer activity of a Schiff base *NNOO* chelate ligand and Pd(II) complex. PLoS ONE 15(4): e0231147. https://doi.org/10.1371/journal.pone.0231147 The publisher apologizes for the error.
