# Supplementary figures and images for: Synthesis, characterization, quantum chemical calculations and anticancer activity of a Schiff base NNOO chelate ligand and Pd(II) complex
Source: PLoS One. 2020 Apr 14;15(4):e0231147. doi: 10.1371/journal.pone.0231147 (PMC7156068; doi:10.1371/journal.pone.0231147)

**1HNMR Spectrum of L2F**


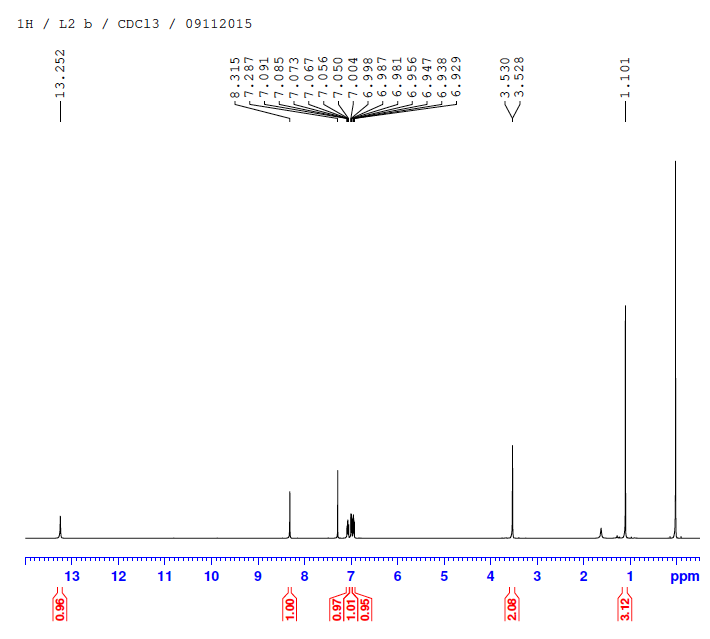


C1-OH

HC7=N

C10-H3

C8-H2

Solvent peak, CDCl3


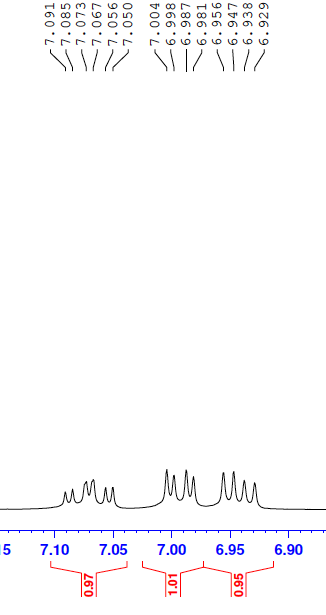


C3-H

C5-H

**1HNMR Spectrum of PdL2F**


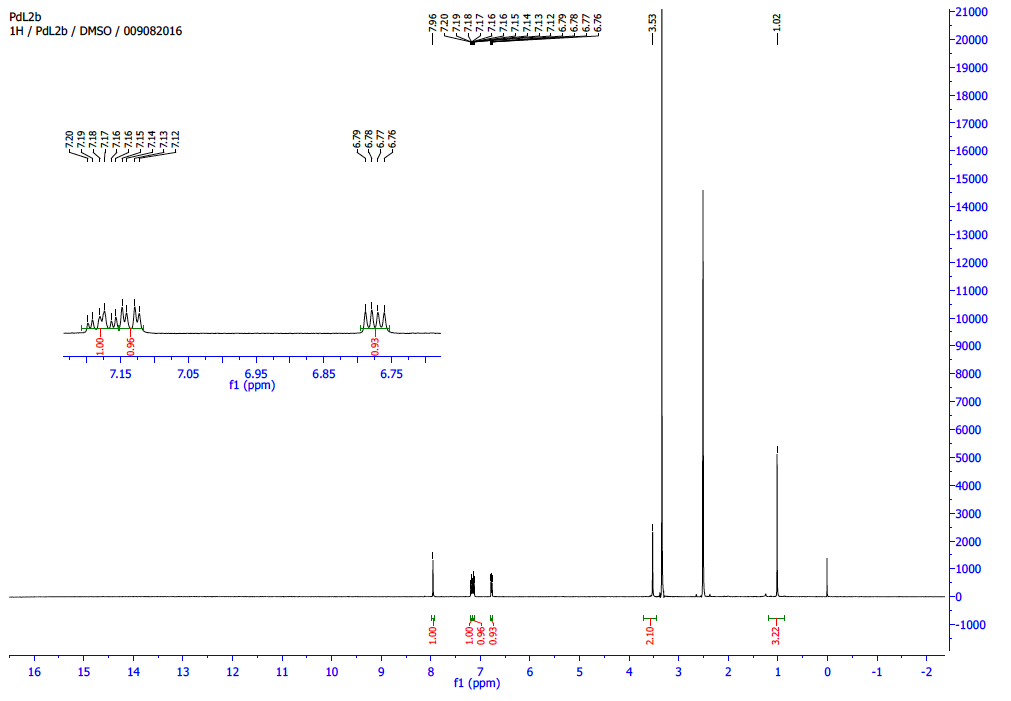

Supplement: S1 Fig — (DOCX) [file pone.0231147.s001.docx]

**13C NMR spectra of L2F**


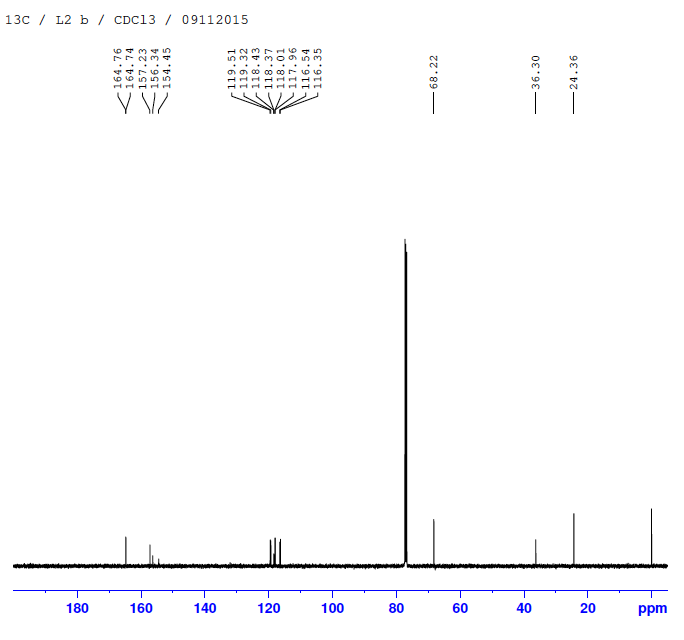


C1-OH

HC7=N

C4-F

C8-H2

C9-(CH3)2

C10-H3

Solvent peak, CDCl3

C6-H

C5-H

C2-H

C3-H

Supplement: S2 Fig — (DOCX) [file pone.0231147.s002.docx]
